# Genital prolapse: epidemiology, clinic and therapeutic at Saint Joseph Hospital of Kinshasa

**DOI:** 10.11604/pamj.2020.37.196.21818

**Published:** 2020-10-29

**Authors:** Antoine Tshimbundu Kayembe, Andy Mbangama Muela, Alex Mutombo Baleka, Dieudonné Sengeyi Mushengezi, Rahma Rachid Tozin

**Affiliations:** 1 Department of Gynaecology and Obstetrics, Faculty of Medicine, University Notre-Dame of Kasayi, Central Kasaï, Democratic Republic of the Congo,; 2 Department of Gynaecology and Obstetrics, Faculty of Medicine, University of Kinshasa, Kinshasa, Democratic Republic of the Congo

**Keywords:** Genital prolapse, epidemiology, clinic, therapeutic, Saint Joseph Hospital, Kinshasa

## Abstract

The aim of the study was to describe the epidemiological, clinical and therapeutical profile of genital prolapse in the gynecology and obstetrics service of Saint Joseph Hospital of Kinshasa. This is a descriptive study carried out from medical files of patients who have suffered from genital prolapse in the gynecology and obstetrics service of Saint Joseph Hospital from January 1^st^, 2008 to December 31^st^, 2017. It is based on the no probabilistic sampling of suitability. We recorded 161 cases of genital prolapses upon 13957 patients. The genital prolapses frequency was 1.2% with an annual average of 16.1 cases (SD 10.1) per year. The symptomatology consisted of pelvic mass associated with urinary and digestives troubles (94.0%, n=140). The stage III of cysto-colpocele was the most frequent (56.0%, n=82). The vaginal hysterectomy associated to rectocele and cystocele cure was the most performed operation (52.0%, n=69). The recurrence rate was of 2.0% (3 out of 148 cases). The genital prolapse really exist in our milieu, its symptomatology is classical and its treatment is mostly surgical by vaginal access.

## Introduction

Genital prolapse is a dynamic disease which can worsen or recede above all in pregnant women in the postpartum period [1, 2]. It comprises a great recurrence risk after surgical treatment [1, 3]. It causes several troubles such as: urinary, digestive and genital problems which hamper the patients [1, 4, 5]. Its diagnosis rest on the clinical and paraclinical exams as perineal ultrasound, cysto-defecography (CDG) and magnetic resonance imaging (MRI) [3, 6]. The treatment for genital prolapse can be medical or surgical [3, 6, 7]. The prevalence of genital prolapse varies from 2.9% to 97.7% in the world according to the method used for the study. It is estimated from 2.9% to 11.4% when the method used is a symptom´s questionnaire [4, 5, 8, 9-12] and from 31.8% to 97.7% when clinical exam with the pelvic organs prolapses quantification (POPQ) is performed [2, 13-17]. In Asia and Africa, this prevalence is not known due to the lack of survey and studies in the general population [18]. In the Democratic Republic of Congo, this prevalence is not known and data to estimate its incidence are inexistent [18]. The lack of epidemiological data of genital prolapse in our milieu pushed us to conduct the present in order to describe the epidemiological, clinical and therapeutical profile of genital prolapses in the gynecology and obstetrics service (GOS) of Saint Joseph Hospital (SJH) of Kinshasa.

## Methods

**Study design and setting:** this descriptive study is based on the no probabilistic sampling of suitability for cases selection. It included the medical files of patients who suffered from genital prolapses and treated in GOS of SJH. All medical files not found and those that contained less than 50% of studied variables were excluded. One hundred and forty-eight available medical files have been included and thirteen were excluded because their medical files have not been found (either in total one hundred sixty-one cases of genital prolapses registered).

**Study population:** the target population comprised all patients who suffered from genital prolapse and admitted in GOS of SJH of Kinshasa from January 1^st^, 2008 to December 31^st^, 2017. The GOS of SJH of Kinshasa were chosen because of the presence of formed medical staff, the frequent contact with the patients who suffer from genital prolapses, and a free management through the fistula care program.

**Data collection:** data were collected from the registries of GOS and the operating rooms, the medical files of patients who suffered from genital prolapses at SJH and the data collection record. The study´s variables are year, clinical characteristic (symptomatology, types and stage of genital prolapses) and therapeutical variables (type of treatment performed, therapeutical complications and evolution).

**Statistical analysis:** data were analyzed using Statistical Package for Social Sciences (SPSS) software version 20. We used the average (SD) to present the quantitative variables and the proportion to present the qualitative variables.

**Ethical considerations:** principles of medical ethics and documentary studies rules have been respected; the data were collected confidentially and treated anonymously.

## Results

**Figure 1:**
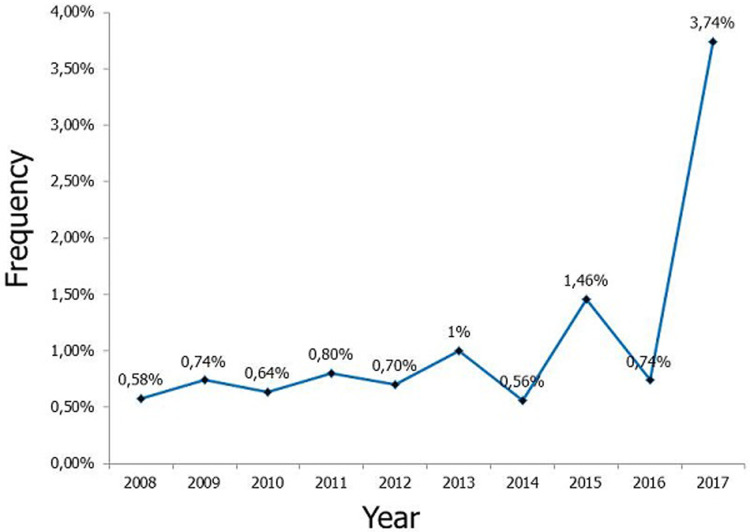
frequency's evolution of genital prolapse during our study period

**Genital prolapses frequency:** we registered 161 cases of genital prolapses out of 13957 patients in the GOS of SJH, resulting in an overall frequency of 1.2%. During the study´s period and according to the Figure 1, this frequency evolved in saw way cog, from 0.6% in 2008 to 3.7% in 2017. The annual average of genital prolapses cases was of 16.1 cases (SD 10.1) per year. We remind that 148 available cases have been included and 13 excluded because their medical files have not been found or were not available (in total 161 cases of genital prolapses registered).

**Clinical characteristics of genital prolapses:** the most frequent complaint was an association of urinary, genital and digestive troubles. The pre-operative symptomatology was made of the sensation of pelvic mass essentially associated to urinary incontinence of stress, imperious micturition with or without incontinence, dysuria, constipation, anal incontinence and dyspareunia (Table 1). The most frequent genital prolapses type was anterior made of the cysto-colpocele essentially. The stage III of genital prolapses was the most frequent in 55.4% of cases.

**Table 1:** pre-operative symptomatology of genital prolapse

Pre-operative symptom	N=148	%
**Urinary**		
Urinary incontinence of stress (UIS)	45	30.40%
Imperious micturition with or without UIS	62	41.90%
Dysuria	37	25.00%
**Digestive**		
Constipation	41	27.70%
Anal incontinence	11	7.40%
**Sexual**		
Dyspareunia and pelvic pain	102	68.90%
Pelvic mass sensation	140	94.59%

**Therapeutical characteristics of genital prolapse:** surgery was the most performed in 89.2% of cases and the vaginal access was most used in 98.6% of cases. Hysterectomy associated to rectocele and cystocele cure was the most performed in 57.0% of cases whereas the most frequent therapeutical complications was the vesical lesions in 2.0% of cases. The post-therapeutical evolution was marked by the disappearance of genital prolapses in 80.0% of cases, its recurrence in 2.0% of cases and its spontaneous regression in 18.0% of cases (Table 2).

**Table 2:** clinical and therapeutical characteristics of genital prolapses

Clinical and therapeutical characteristics	N= 148	%
**Type of genital prolapses**		
Anterior (cysto-colpocele)	52	35.1
Middle (hysterocele)	15	10.1
Posterior (rectocele)	3	2.0
Association of anterior and middle genital prolapses	29	19.6
Association of anterior and posterior genital prolapses	5	3.4
Association of middle and posterior genital prolapses	15	10.1
Association de 3 genital prolapses types	29	19.6
**Stades of genital prolapses**		
Stage I	0	0.0
Stage II	42	28.4
Stage III	82	55.4
Stage IV	24	16.2
**Surgery**	132	89.2
Hysterectomy	10	8.3
Hysteropexia	4	3.3
Cystocele cure	46	28.9
Rectocele cure	3	2.5
Association	69	57.0
**Vaginal access**	130	98.6
**Complications**		
present (vesical lesions)	3	2.3
Absent	145	97.7
**Post-therapeutical evolution**		
Recurrence of genital prolapses	3	0.7
Disappearance of genital prolapses	119	81.8
Regression	26	17.6

## Discussion

The frequency of genital prolapses was of 1.2% at SJH. Its evolution was in a saw cog way from 0.6% in 2008 to 3.7% in 2017 during the period of our study. The high frequency observed in 2017 was linked to the first public awareness campaign for the free treatment of genital prolapses at SJH. Our frequency is lower than those found by the Hamri *et al*. study in Morocco [19], Seven *et al*. in Turkey [20] and Rodrigues *et al*. in Brazil [21] which were of 2.4%, 5.6% and 7.5% respectively. It is almost identical to those found by Kishawas *et al*. at Bangladesh (1.1%) [22], Alherrech *et al*. in Morocco (1.1%) [23] and Zhu *et al*. in China (1.2%) [24]. It is higher than 0.5% of Fanny *et al*. in Ivory Coast [25]. This frequency difference can be explained by the single institution character of our study and the more or less free treatment of genital prolapses in the account of fistula care. The most frequent complaint at consultation was an association of urinary, digestive and genital troubles in 97.3% of cases and the preoperative symptomatology was made of sensation of pelvic mass associated to imperious micturition with or without UIS in 41.9%, urinary incontinence of stress (UIS) in 30.4%, constipation in 27.0%, dysuria in 25.0% and anal incontinence in 7.4%. Our results were in accordance with those found by Rivaux *et al*. [26]. These symptoms are due not only to anatomical anomaly of pelvic fascia and anal levator secondary to direct traumatic lesions or to innervation abolition at the roof of the pelvic floor´s weakening, but also to high intra-abdominal pressure (≥6mmHg) with high intra-vesical pressure [27-29].

The anterior genital prolapses which is made of the cysto-colpocele was the most frequent type of genital prolapses: isolated in 35.0% of cases and associated to middle genital prolapses in 20.0%, to posterior genital prolapses in 10.0% and to two precedent in 20.0%. Our results corroborate those of Amblard *et al*. [30], Handa *et al*. [2], Blain *et al*. [16] and Hamri *et al*. [24]. Our types sequence of genital prolapses was cystocele-hysterocele-rectocele, which is similar to some other African studies [18, 19, 23, 25]. This shows that the type sequence of genital prolapses is the same in Africa whereas elsewhere authors reported a different sequence made of cystocele-rectocele-hysterocele [2, 16, 17]. This differences in sequence are based on the epidemiological arguments or observations. As for the stage of genital prolapses, the stage III was the most frequent in 56.0% whereas the stage I was absent. Our results meet those of Thomin *et al*. [31] and Maadi *et al*. [32]. According to Handa *et al*. the advanced stages of genital prolapses had a long term bad prognosis because of their multiple symptom which push the patients to consult whereas the stage I is not symptomatic and do not necessitate treatment [19]. The absence of stage I in our study explains the symptomatic character of our genital prolapses and would be due to its easy regression to stage 0 or its weak progression toward stages II and III as noted by many authors [2, 33].

The surgical treatment was the most practiced in 89.2%. Our results are in accordance with Boulanger *et al*. [34, 35]. According to Villet *et al*. and De Tayrac *et al*. surgical treatment in the genital prolapses is ideal because it allows to correct the anatomical degradation without causing the new troubles [36, 37]. This must be the purpose of surgery performed in our milieu. The rate of pessary´s usage is of 0% in our cases series whereas it´s more than 11.0% in many studies [35, 38, 39]. This can be explained by the policy in our milieu to favour the surgical reparation in all the patient´s in good health as Boulanger *et al*. observed it [34, 35]. According to Clemons *et al*., the high number of exteriorized genital prolapses was very difficult to control, with the pessary and the long-term complications linked to the pessary´s usage, it prevented its usage [40]. These two arguments can also explain our null rate of pessary´s usage.

The vaginal surgical access was predominant with 98.0%. Our results meet De Tayrac *et al*. [36]. According to Boulanger *et al*. and Maher *et al*., the vaginal surgical access has the advantage of operative duration smaller than abdominal surgical access and of a faster resumption of activities [34, 35, 41]. These two advantages can explain our high rate of surgery by vaginal access. The laparoscopic access hasn´t been used in our cases series because of the absence of laparoscopy´s equipment and of formed medical staff. As for the surgical types, the hysterectomy associated to cystocele and rectocele cure was the most performed. This can be explained by the high percentage of our genital prolapses cases which associated the three types: anterior, middle and posterior. Our results rejoin Hamri *et al*. [19] and Alharrech *et al*. [23]. According to Boulanger, the hysterectomy which is performed in the prolapses cases must be associated to the correction´s gesture of prolapse, and the uterine conservation is realized only if it doesn´t exist the uterine anomalies which are discovered to cervical smear, to endometrium biopsy and to systematic pelvic ultrasound [34, 35]. Unfortunately, these systematic pre-operative exams weren´t realized in our milieu because of not only ignorance of practitioners but low social and economic level of our population.

In the study of Korahanis *et al*., the fixing of trans-vaginal prosthesis in polypropylene associated to genital prolapses cure was the most performed in 80.0% [42]. This is not the case in our study because of the unavailability of prosthesis. Per-operative complications were present in 2.0% (vesical lesions) whereas post-operative complications were missing. Our results are smaller than De Tayrac *et al*. which reported an overall rate of per-operative complications of 5.8% [36]. The difference in complications rates can be explained by the surgical techniques mastery of genital prolapses treatment by the practitioners, despite the equipment´s poverty. In the study of Hefni *et al*. and of Lovartsis *et al*., the post-operative complications was rare [43, 44]. These results are almost similar to ours and express the best cares quality. Concerning the post-therapeutical evolution, the recurrence rate was of 2.0%. Our results are smaller than those of many authors who reported a recurrence rate higher than 30.0% [36, 45-48]. The difference of recurrence rates can be explained by the single hospital character of our study and the surgical techniques mastery by our practitioners. Weakness of our study is the no evaluation of the genetic factors impact upon the genital prolapses appearance and its strength is to be the first to study the particularity of epidemiology, clinical and therapeutical characteristic of genital prolapses in the hospital milieus of Kinshasa.

## Conclusion

It appeared in this study clearly that, the genital prolapses really exists with a frequency of 1.2% with symptomatology almost classical and made of pelvic mass associated to urinary and digestive troubles (94.0%), the cysto-colpocele of stage III is the most frequent type (56.0%), the hysterectomy associated to rectocele and cyctocele cure by vaginal access is the most performed (52.0%) with a recurrence rate of 2.0%. Our results are the base of deepened studies upon genital prolapses and they allow the scientists awareness upon genital prolapses studies and the improvement of its treatment in our milieu.

### What is known about this topic

- The genital prolapse is the dynamic disease which can worsen or recede above all in pregnant women during the postpartum period;
- It comprises a great recurrence’s risk after surgical treatment and it causes urinary, digestive and genital problems which hamper the patients;
- The absence of epidemiology, clinic and therapeutic of genital prolapse in hospitals in Kinshasa, DR Congo.

### What this study adds

- The genital prolapses frequency is 1.2%, the cysto-colpocele of stage III is the most frequent type and treated surgically with the recurrence rate of 2.0% in Kinshasa (DRC): this is the base on deepened studies upon genital prolapses in hospital of Kinshasa in DR Congo;
- Scientists will gain awareness upon genital prolapse’s studies and to improve its treatment in our milieu.
